# Pilot Testing and Implementation of a mHealth tool for Non-communicable Diseases in a Humanitarian Setting

**DOI:** 10.1371/currents.dis.e98c648aac93797b1996a37de099be74

**Published:** 2017-06-05

**Authors:** Shannon Doocy, Kenneth Paik, Emily Lyles, Hok Hei Tam, Zeina Fahed, Eric Winkler, Kaisa Kontunen, Abdalla Mkanna, Gilbert Burnham

**Affiliations:** Department of International Health, Johns Hopkins Bloomberg School of Public Health, Baltimore, MD, USA; Massachusetts Institute of Technology Sana mHealth Group, Cambridge, MA, USA; Department of International Health, Johns Hopkins Bloomberg School of Public Health, Baltimore, MD, USA; Massachusetts Institute of Technology Sana mHealth Group and Department of Chemical Engineering, Cambridge, MA, USA; International Organization for Migration, Beirut, Lebanon; Massachusetts Institute of Technology Sana mHealth Group, Cambridge, MA, USA; International Organization for Migration, Beirut, Lebanon; International Organization for Migration, Beirut, Lebanon; Department of International Health, Johns Hopkins Bloomberg School of Public Health, Baltimore, MD, USA

## Abstract

**Introduction.:**

Given the protracted nature of the crisis in Syria, national and international assistance agencies face immense challenges in providing for the needs of refugees and the host Lebanese due to the high burden of noncommunicable diseases (NCDs) among both populations. These are complex conditions to manage, and the resources for refugee care limited, having dramatic implications for Lebanon’s health system.

**Methods.:**

A longitudinal cohort study was implemented from January 2015 through August 2016 to evaluate the effectiveness of treatment guidelines and an mHealth application on quality of care and health outcomes for patients in primary health care facilities in Lebanon serving Syrian refugees and host communities.

**Results.:**

Overall, reporting in clinic medical records remained low, however, during the mHealth phase recording of BMI and blood pressure were significantly greater in the mHealth application as compared to clinic medical records. Patient exit interviews reported a much more frequent measurement of weight, height, blood pressure, and blood glucose, suggesting these may be assessed more often than they are recorded. Satisfaction with the clinic visit improved significantly during implementation of the mHealth application as compared to both baseline and guidelines implementation in all measures. Despite positive changes, provider uptake of the application was low; patients indicated that the mHealth application was used in a minority (21.7%) of consultations. Provider perspectives on how the application changed patient interactions were mixed.

**Discussion.:**

Similar to previous evidence, this study further demonstrates the need to incorporate new interventions with existing practices and reporting requirements to minimize duplication of efforts and, consequently, strengthen provider usage. Additional research is needed to identify organizational and provider-side factors associated with uptake of similar applications, particularly in complex settings, to optimize the benefit of such tools.

## Introduction

Since the outset of the Syrian conflict in March 2011, an estimated 4.6 million Syrians have fled to neighboring countries.^1^ With over 1.1 million Syrian refugees registered with the United Nations High Commissioner for Refugees (UNHCR) as of January 2017, Lebanon is host to the highest number of refugees per capita in the world.^2^ Health assistance for Syrian refugees is based on the primary healthcare strategy and coordinated by UNHCR and the Lebanese Government. Syrian refugees can utilize primary healthcare services at subsidized rates in select existing primary healthcare centers and primary level facilities across Lebanon. Public sector care for Syrian refugees and vulnerable Lebanese based on routine care in primary health facilities with referral to secondary and tertiary care for management of difficult cases and complications.

Type 2 diabetes prevalence has been estimated at 7.4% in Syria and 14.4% in Lebanon.^3^ Previous literature has estimated regional prevalence of hypertension at 29.5%, prevalence in Syria at 24.9%, and 28.8% in Lebanon.^4^^,^^5^^,^^6^ National and international assistance agencies face immense challenges in providing for the needs of affected populations in both refugee and host communities due to the high burden of noncommunicable diseases (NCDs) among refugees and the host country population. These are complex conditions to manage, and the resources for refugee care limited.^7^^,^^8^^,^^9^ Intervention through mobile technologies (mHealth) has become increasingly common in recent years, and have shown promise in the potential for technology to leverage the widespread adoption of mobile devices and overcome infrastructure limitations in low-resource or complex settings.^10^^,^^11^ Such interventions use mobile devices to improve public health across a wide range of domains with applications including but not limited to client education, point-of-care diagnostics, electronic health records and decision support, patient-provider communication, provider training and education, etc.^12^

In consideration of this, we undertook a study to evaluate the effectiveness of treatment guidelines and an mHealth application on quality of care and health outcomes in primary care settings in Lebanon. Findings related to the mHealth application, including quality of the care-seeking interaction, patient satisfaction and provider perceptions, are presented along with discussion of potential deployment of mHealth applications for use in hypertension and diabetes care in humanitarian settings.

## Methods

A longitudinal cohort study was implemented from January 2015 through August 2016 in primary health facilities in Lebanon that serve both Syrian refugees and Lebanese. Its two research aims were: (i) to develop, adapt, and test existing standards and guidelines for treatment, including counseling, for persons with hypertension and type 2 diabetes (or both); and (ii) to evaluate the effectiveness of an mHealth tool. Standard best-practice guidelines were adapted to the local context taking into account national protocols, prescribing practices, and the primary care context where they would be applied. Providers were subsequently trained in use of the guidelines and provided with written materials to support clinical decision making.^13^^,^^14^^,^^15^ The mHealth application included a patient-controlled health record (PCHR) and served as an electronic medical record as well as a decision support tool for providers. The mHealth tool has the potential to improve quality and continuity of care, health literacy, and health outcomes for patients. Providers were trained in use of the application and support provided to health facilities for its implementation.^16^ The study used a phased introduction of the two interventions over 20 months with longitudinal measurement of outcomes. The clinics continued to use their standard record systems which were either paper or electronic.

Participants consisted of patients at ten health care centers in Lebanon supported by the International Organization for Migration (IOM) and International Medical Corps (IMC) in the South (n=3), Bekaa (n=3), and Beirut/Mount Lebanon (n=4) governorates (Figure 1). Patients at these locations were predominantly Lebanese and Syrian refugees. Individuals without a diagnosis of hypertension or type 2 diabetes, those less than 40 years of age, and adults lacking capacity to independently participate in interviews were excluded.

**Figure:**
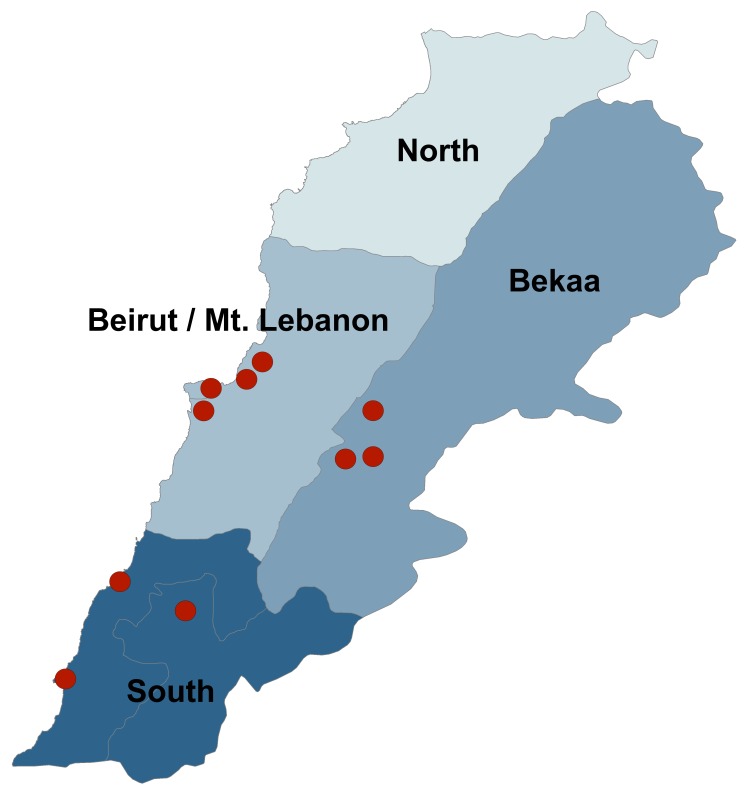
Figure 1. Participating Primary Health Centers by Geographic Area

A total of 1020 participants were enrolled and 793 (78%) completed the study. Sample size calculations were based on the proportion of providers that adhere to treatment guidelines, and assumed a baseline rate of 50% for adherence to guidelines (the most conservative rate that would ensure the ability to detect significant differences from all other rates). This is a reasonable assumption given that proposed guidelines did not differ substantially from other best practice guidelines, thus patients could be on recommended treatment at baseline. Sample size calculations were performed using Stata 13, assumed α=0.05, β=0.20 (power=0.80) and were one-sided based on the assumption that quality of care will not decrease because of the intervention. The final sample of 793 participants was sufficient to detect increases ≥5.0% for provider adherence to guidelines.

This study was designed using a mixed method approach with qualitative and quantitative data collected throughout. Patients were recruited at clinics and if they indicated willingness to participate, a follow up phone call was made. This verified consent and a baseline interview collected information on demographic characteristics; medical history and recent care seeking behaviors; and knowledge, attitudes, and practices related to type 2 diabetes and/or hypertension. Following enrollment, medical record reviews were also conducted for each patient recording information related to provider compliance with guidelines and quality of care; frequency of visits; generic patient outcomes (death and loss to follow-up), and disease-specific patient outcomes (complications and adverse events of hypertension and type 2 diabetes). Phone interviews and record reviews were repeated at the end of each study phase (guidelines, mHealth). In addition, a subset of patients were telephoned after facility visits to complete a brief exit interview. Both patients and providers participated in focus group discussions at the end of guidelines (providers only) and mHealth application phases.

Data were collected on tablets using the Magpi mobile data platform by DataDyne LLC (Washington, DC) and analyzed using Stata 13 (College Station, TX) using descriptive statistics and standard methods for comparison of means and proportions. Uptake of the mHealth application was low; a total of 154 records were extracted from the application dataset whereas a total of 878 record reviews were completed in the mHealth phase. Differences in patient characteristics and phases were examined using chi-square and t-test methods. An immediate form of two-sample tests of proportions was performed using the Stata ‘prtesti’ command to determine whether the proportions in the mHealth app and paper records were statistically different. Focus group data were analyzed using qualitative description and content analysis.^17^

Ethics approval for this study was obtained from the Ministry of Public Health in Lebanon and Institutional Review Board at The Johns Hopkins Bloomberg School of Public Health.

## Results

**Quality of the Clinical Interaction.** Completeness of reporting of clinical measurements in medical records was one metric used to assess the mHealth application. There was a statistically significant increase of 7.7% (CI: 4.6-10.8, p<0.001) in the proportion of patients with BMI reported in the mHealth phase as compared to the guidelines phase. However, following adoption of the mHealth application there were no significant changes in the proportion of hypertension patients that had blood pressure readings recorded (p=0.241) or diabetes patients that had blood sugar tests reported (p=0.297) (Figure 2). Overall, reporting in clinic medical records remained low, however, during the mHealth phase recording of BMI and blood pressure were significantly greater in the mHealth application as compared to clinic medical records (BMI: 47.4% vs 15.8%, p<0.001; BP: 74.5% vs. 40.7%, p<0.001); there was no significant difference in reporting of blood glucose test results (39.9% vs 34.0%, p=0.185). Patient exit interviews reported a much more frequent measurement of weight (67.8%), height (65.6%), blood pressure (88.9%), and blood glucose (79.1%), suggesting these parameters may be assessed more often than they are recorded. Quality of patient-provider interactions was assessed based on patient exit interviews conducted via phone within two weeks of the appointment. Statistically significant change was detected in all four measures (provider inquiry of medical history, complications with prescribed medication, prompting for questions from the patient, and recommending follow-up or referral care). Similarly, for lifestyle counseling, significant increases in the proposition of patients reporting that various types of lifestyle counseling given by health providers was observed in the mHealth phase (Figure 3).

**Figure:**
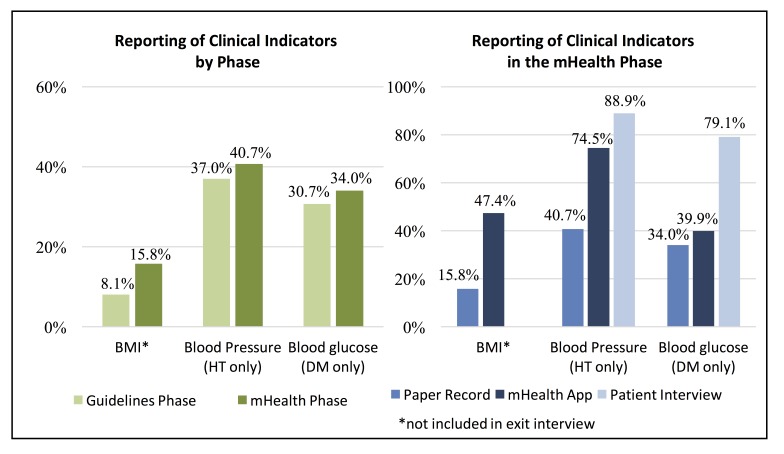
Figure 2. Reporting of Clinical Indicators by Phase and Information Source in the mHealth Phase

**Figure:**
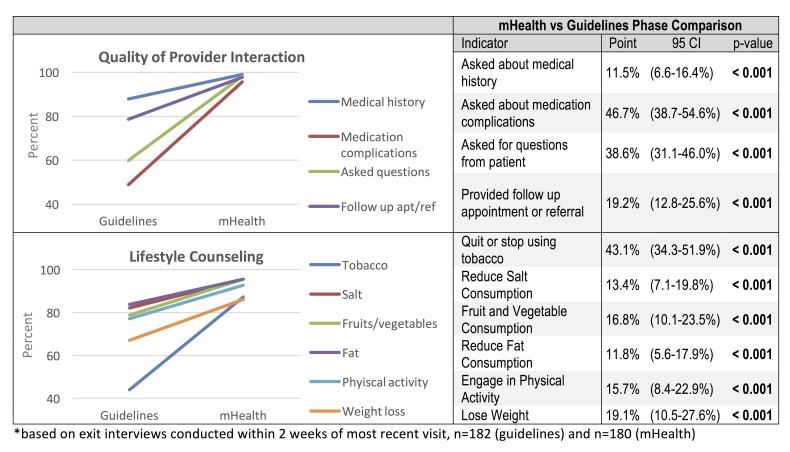
Figure 3. Change in Provider Interaction and Lifestyle Counseling between Phases*

Despite positive changes in the quality of patient provider interactions and frequency of lifestyle counseling during the mHealth phase, patients indicated that the mHealth application was used in a minority (21.7%, CI: 15.9-28.4) of consultations (Table 1). While it is possible that the application was used after consultation, as was reported by providers, the intended use of the application during the consultation as a decision support tool occurred infrequently according to patients. Receipt of printed information on current medication and lifestyle modification (outputs of the mHealth application) was reported by 48.7% (CI: 32.4-65.2%) and a summary health record by 23.1% (CI: 11.1-39.3%) of patients, indicating application use may have been greater than the 21.7% of patients that recalled observing the provider used a tablet during the consultation.

**Figure:**
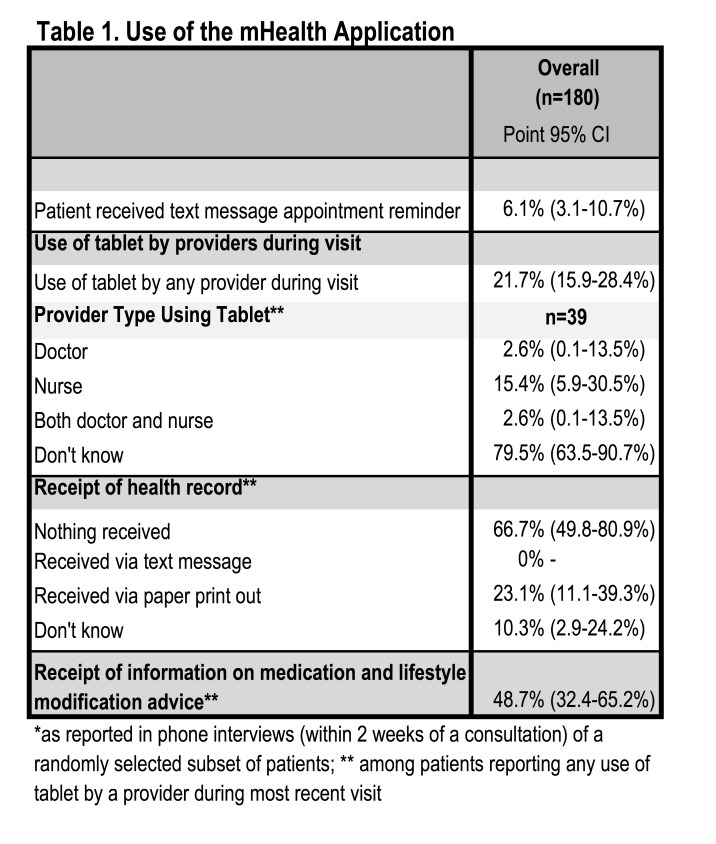
Table 1. Use of the mHealth Application

**Patient Satisfaction.** Patient satisfaction with health workers and clinical interactions was assessed based on patient exit interviews conducted via phone within two weeks of the appointment. Satisfaction with the clinic visit improved significantly during implementation of the mHealth application as compared to both baseline and guidelines implementation in all measures (Table 2). All patients agreed with the statement “I trust in the skills and abilities of the health workers” during implementation of the mHealth application, an 11.0% increase from 89.0% at baseline (p=0.001) and 3.9% increase from the guidelines implementation phase (p=0.007). Nearly all patients (98.9%) believed that health workers “did a good job explaining [their] illness” during the mHealth phase, a significant increase from 68.0% at baseline and 86.0% during guidelines implementation (increases of 30.9% and 12.9%, respectively; p=0.001). Similar changes were observed in patients’ belief that health workers “did a good job explaining [their] treatment” in the mHealth phase with 98.9% of patients reporting satisfaction in the mHealth phase as compared to 68.0% at baseline (30.9% increase, p=0.001) and 83.2% during guidelines implementation (15.6% increase, p=0.001). Patients’ perceived ability to discuss problems or concerns about their condition with the health workers was also reported by nearly all respondents during the mHealth phase (98.9%), significantly higher than at baseline (69.0%; 29.9% increase; p=0.001) and guidelines implementation (74.3%; 24.6% increase; p=0.001). Most patients (95.0%) felt they were involved in the consultation and treatment decisions during the mHealth phase, significantly greater than at baseline (58.0%; 37.0% increase; p=0.001) and during guideline implementation (79.3%; 15.7% increase; p=0.001). Satisfaction with the amount of time spent with health workers was also reported by nearly all patients in the mHealth phase (97.2%), a significant increase of 36.2% from 61.0% at baseline (p=0.001) and 9.0% increase from 88.3% during guidelines implementation (p=0.001).

**Figure:**
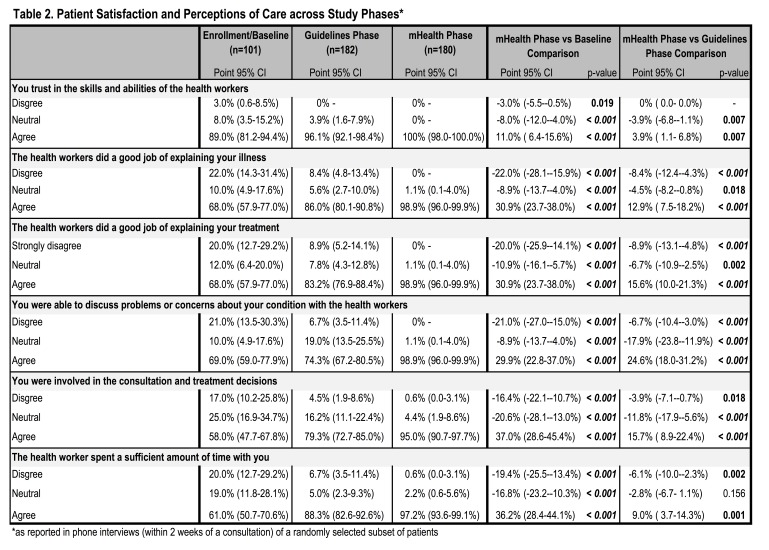
Table 2. Patient Satisfaction and Perceptions of Care across Study Phases*

**Provider Perceptions. **Provider perspectives on how the application changed patient interactions were mixed. In some locations, the application was perceived as extra work or time consuming and provider attitudes were negative whereas in other places provider attitudes were positive and perceived benefits included improved patient understanding of lifestyle behaviors and medications, and greater patient satisfaction when the application was used. Providers noted the evidence-based standardized treatment algorithms, automated determination of BMI and risk category, the patient printouts, and ability to view the records across facilities as the main benefits to using the application. However, they indicated that gains in the ability to track patient information across time were minimal. The reasons given for this included the short evaluation period, where patients may not have returned for a follow up visit, and use of other electronic medical record platforms in some facilities.

Providers noted that using the application was time consuming and poor wireless connectivity sometimes made it difficult for patients to receive the printouts. Other reported challenges were technical glitches in the application and the fact that it was not integrated with existing systems. Recommendations to improve the utility of the application included addressing technical glitches, linking the application to existing electronic records systems, considering a dedicated staff member to use the application or dedicated clinic hours for patients with hypertension and diabetes, and expanding the lifestyle education component and the amount of detail included in patient print outs. Providers in clinics with electronic medical records systems were less inclined to continue use of the application than those in facilities with paper records but there was consensus that ongoing technical support would be required.

## Discussion

The rapid expansion of mHealth interventions over the past decade has been justified by the immense potential of technology to overcome infrastructure limitations and leverage the widespread adoption of mobile devices.^10^,^11^ Existing evidence of the efficacy, effectiveness, and implementation factors of mHealth interventions has largely been inconclusive and mixed, in part due to inadequate study design.^18^^,^^19^ As might be anticipated, there is limited evidence on the advantages of mHealth tools for NCDs in humanitarian settings. The strongest evidence, from a United Nations Relief and Works Agency for Palestine Refugees (UNRWA) pilot program aimed at addressing NCD treatment challenges, demonstrated that use of electronic medical records resulted in improved quality and continuity of care for diabetes and hypertension patients.^20^^,^^21^ A recent review of health interventions in humanitarian crises concluded there was minimal evidence for interventions for the most prevalent NCDs and recommended expanding the evidence base on interventions for common NCDs, such as hypertension and diabetes.^22^

The need to address NCD prevention and control in humanitarian settings is recognized, but there is little evidence to guide practice and policy.^22^ Knowledge exists around what interventions are effective, but there are gaps in the adaptation of protocols and guidelines to different crisis settings and in the implementation of NCD interventions.^22^ The global World Health Organization-International Telecommunication Union (WHO-ITU) mHealth initiative to promote the use of mHealth in the prevention, treatment, and control of NCDs could be applied in humanitarian settings to further strengthen the evidence base for intervention in these contexts.^23^ Current best practices include careful consideration for interoperability and open standards of mHealth tools as well as coordination with local governments, partners, and implementers. Previous evidence has shown that the added benefit of mHealth tools are most successful when providers and patients adhere to accepted guidelines and practices and are compliant with treatment prior to implementation. In these cases, mHealth tools may serve as a catalyst for improving health outcomes.^24^ However, as the authors of one review of mHealth tools for NCD treatment and management observe, “mHealth tools are communication platforms and delivery mechanisms, not solutions in and of themselves.”^24^

Difficulties in developing and deploying new technologies, including running repairs on technical glitches and challenges with initial programming and rollout/uptake of the application lessened usage by clinicians. Some providers initially interested in the application were disappointed by technical problems and frequent crashes in initial versions of the application, leading them to use the application less frequently over time. Low uptake persisted among some providers despite technical repairs and supportive supervision by study staff in health facilities. In the case of the Lebanon pilot, limited use of the application was related more to provider attitudes than objective usability or benefits. Providers conveyed numerous reporting requirements from the health facility itself, the MoPH, and supporting organizations. Many of these documentation requirements were perceived as duplicative and demanding on health providers’ time, a finding similarly observed in previous studies of similar mHealth interventions.^24^ The application was perceived as more beneficial in facilities that did not previously have electronic records/reporting. Incorporating an mHealth tool in a manner that facilitates streamlined reporting to fulfill the requirements of all necessary reporting is likely to improve uptake and complete reporting. The need for organizational changes to support feasibility and facilitate uptake of facility level mHealth interventions noted in previous studies similarly applies to the Lebanon pilot.^25^ Additionally, in Lebanon much of the care for persons with hypertension was provided by cardiologists and for diabetes by endocrinologists. In a location where patients with these conditions are treated by primary health personnel, the electronic treatment guidelines could have a greater ability to guide treatment, and consequently a greater uptake.

Elements of the mHealth application piloted in this study can be considered as focusing on “mAdherence” or the use of mHealth tools in improving patient adherence to chronic disease management. Examples of elements that may be included in mAdherence tools that have been previously examined include automated reminders, text messages with educational and motivational content, healthy living challenges, and wireless transmission of data which have been shown to contribute to increased self-care awareness and knowledge about chronic diseases.^26^^,^^27^^,^^28^^,^^29^ Portability of patient records across facilities, an initial goal of this application, has minimally been studied in the past, though is a strong advantage of PCHRs in areas with fluid population movement. Most previous mAdherence studies focus on outcomes related to patients as the end-user; far fewer studies address provider acceptability or related provider-side uptake factors.^24^ Those that have looked into provider-side factors identified similar implementation concerns as this study including, but not limited to increased workload, supervision needs, sound technical design, and usability.^24^^,^^30^ However, a 2008 study of electronic health record implementation in five provider organizations in the U.S. indicated that despite providers’ initial concerns that the process of implementing electronic health records would obstruct workflow, findings suggest that electronic health record implementation actually improved workflow by enhancing accessibility of patient information across clinics and systematizing staff communications.^31^

**Limitations.** Comparison of completeness of reporting across study phases may have underestimated changes in patient and provider practices in the guidelines and mHealth phases where all available information in the patient record was included at baseline, regardless of what was recorded at the most recent visit. Simultaneous development and introduction of the application led to frustration among users when the application did not perform as expected, which reduced provider enthusiasm. Another barrier to uptake was multiple reporting requirements and electronic record systems, which led to the perception that the application was redundant (despite dissimilarities to existing systems in most cases). While the completion rate was relatively high (78%), expired or changed phone numbers were the greatest contributor to incomplete patient follow-up. Population movement within or outside of Lebanon also contributed to a lesser extent to loss to follow-up (n=19). Given the older age and poor health status of enrolled patients inherent with study inclusion criteria, 17 patients (1.7% of enrolled patients) were deceased between enrollment and the end of the study period, though this was not necessarily as a result of conditions of interest in the study. Finally, including patients and providers from only ten health facilities limits representativeness of findings and results may not be generalizable to elsewhere in Lebanon or other settings.

## Conclusion

The difficulties in developing and implementing facility level mHealth interventions in complex contexts are furthered by a paucity of evidence of provider acceptability and provider-side uptake factors. Evidence demonstrates the necessity of incorporating new interventions with existing practices and reporting requirements to minimize duplication of efforts and, in turn, strengthen provider usage. However, further research is needed to identify organizational and provider-side factors associated with uptake of similar applications, particularly in less developed and complex settings, including humanitarian contexts. A more robust evidence base for implementation of such tools is needed to maximize their benefit.

## Corresponding Author

Shannon Doocy, Johns Hopkins School of Public Health, 615 N Wolfe St, Baltimore MD 21205. Tel. +1 410 502 2628. Email: doocy1@jhu.edu

## Competing Interests

Shannon Doocy is a member of the PLoS Currents Disasters Editorial Board. The authors have no other competing interests.

## Data Availability

Minimal underlying data for this manuscript is deposited publicly and can be accessed through the following reference: Doocy, Shannon, 2017, "mHealth tool for NCDs in Lebanon (app adoption analysis)", doi:10.7910/DVN/WU9LBZ<http://dx.doi.org/10.7910/DVN/WU9LBZ>, Harvard Dataverse, V1.

## Funding Statement

This research was funded by Research for Health in Humanitarian Crisis (R2HC). The funding body had no role in the design or implementation of the research and did not participate in analysis or presentation of findings.
